# Andrographolide Inhibits ER-Positive Breast Cancer Growth and Enhances Fulvestrant Efficacy *via* ROS-FOXM1-ER-α Axis

**DOI:** 10.3389/fonc.2022.899402

**Published:** 2022-05-09

**Authors:** Tong Xu, Yanyu Jiang, Shuying Yuan, Li Zhang, Xihui Chen, Weili Zhao, Lili Cai, Biying Xiao, Lijun Jia

**Affiliations:** Cancer Institute, Longhua Hospital, Shanghai University of Traditional Chinese Medicine, Shanghai, China

**Keywords:** andrographolide, breast cancer, ROS, ER-α, fulvestrant, bioinformatics analysis

## Abstract

Estrogen receptor (ER)-positive breast cancer is the main subtype of breast cancer (BRCA) with high incidence and mortality. Andrographolide (AD), a major active component derived from the traditional Chinese medicine *Andrographis paniculate*, has substantial anti-cancer effect in various tumors. However, the antitumor efficacy and the underlying molecular mechanisms of AD on ER-positive breast cancer are poorly understood. In the present study, we demonstrated that andrographolide (AD) significantly inhibited the growth of ER-positive breast cancer cells. Mechanistically, AD suppressed estrogen receptor 1 (*ESR1*, encodes ER-α) transcription to inhibit tumor growth. Further studies revealed that AD induced ROS production to down-regulate FOXM1-ER-α axis. Conversely, inhibiting ROS production with N-acetylcysteine (NAC) elevated AD-decreased ER-α expression, which could be alleviated by FOXM1 knockdown. In addition, AD in combination with fulvestrant (FUL) synergistically down-regulated ER-α expression to inhibit ER-positive breast cancer both *in vitro* and *in vivo*. These findings collectively indicate that AD suppresses *ESR1* transcription through ROS-FOXM1 axis to inhibit ER-positive breast cancer growth and suggest that AD might be a potential therapeutic agent and fulvestrant sensitizer for ER-positive breast cancer treatment.

## Introduction

Breast cancer is the most commonly diagnosed cancer among women worldwide, which has surpassed lung cancer as the leading cause of global cancer incidence with 11.7% of all cancer cases in 2020 (1). Based on its receptor status, breast cancer can be classified into estrogen receptor (ER)-positive, progesterone receptor (PR)-positive, human epidermal receptor 2 (HER2)-amplified, and triple-negative breast cancer which is lack of the former three biomarkers (2). Among these types, ER-positive subtype represents approximately 75% of all breast cancer cases (3). Therefore, discovering drugs to target estrogen and its receptors, known as endocrine therapy, is an important research topic for ER-positive breast cancer treatment.

Among several endocrine therapy drugs, fulvestrant, a selective estrogen receptor down-regulator, is the only one approved for postmenopausal women with ER-positive breast cancer who failed to respond to tamoxifen as the first- or second-line treatment (4). Clinical studies have reported that fulvestrant significantly improved patients’ progression free survival (PFS) with well tolerated (5, 6). Furthermore, the first-line choice *via* using fulvestrant was associated with greater clinical benefit rate (CBR) than using anastrozole (7). However, the effect of fulvestrant on overall survival (OS) and PFS in ER-positive advanced breast cancer remains to be improved (8, 9).

In recent years, searching for new anti-tumor active substances from natural products of Chinese herbal medicine has become a research hotspot (10). Some natural plant compounds such as paclitaxel and irinotecan have been widely used in breast cancer treatment (11, 12). Andrographlide (AD), one of the major active components of the traditional Chinese medicine *Andrographis paniculate*, has been approved by Food and Drug Administration (FDA) and widely used in clinic to treat inflammatory disease and upper respiratory tract infections (13, 14). In our previous study, we had found that AD induced apoptosis by activating the ATF4-Noxa axis in lung adenocarcinoma cells (15). However, the antitumor efficacy and the underlying molecular mechanisms of AD on ER-positive breast cancer are poorly understood.

In the present study, we demonstrated that AD suppressed *ESR1* transcription through ROS-FOXM1 axis to inhibit the proliferation of ER-positive breast cancer both *in vitro* and *in vivo*. Importantly, we further identified that AD could serve as an effective approach to improve fulvestrant efficacy in ER-positive breast cancer *via* synergistically down-regulating ER-α expression.

## Materials and Methods

### Cell Culture and Reagents

All cell lines used in this study were obtained from the Type Culture Collection of the Chinese Academy of Sciences (Shanghai, China). These cells were cultured in Dulbecco’s Modified Eagle’s Medium (DMEM, BasalMedia, Shanghai, China) which contains 10% fetal bovine serum (FBS, Biochrom AG, Berlin, Germany) and 1% penicillin–streptomycin solution (BasalMedia, Shanghai, China) at 37°C with 5% CO_2_. The cells were exposed to 1 nM β-Estradiol (E2, Sigma-Aldrich, Darmstadt, Germany) and the indicated drugs. Andrographolide (AD) was purchased from Selleck (Houston, Texas, USA). Fulvestrant (FUL) was purchased from MCE (Shanghai, China).

### Cell Proliferation Assay

Cells were seeded in ATPlite plates with 3000 cells per well, in triplicate, and cultured overnight. Cells were treated with the indicated drugs for 72 h, followed by the ATPlite luminescence assay (BD Pharmingen, Franklin Lakes, New Jersey, USA), according to the manufacturer’s instructions. The combination index (CI) was calculated using CompuSyn software in the combination therapy assay (16). CI<1, = 1, and >1 indicate synergism, additive effect, and antagonism, respectively.

### Cell Clonogenic Assay

Cells were seeded in six-well plates (800 cells per well) in triplicate and cultured overnight. Cells were treated with the indicated drugs for 2 weeks. Representative results of three independent experiments with similar trends are presented.

### Isolation of Nuclear and Cytoplasmic Extract

Cytoplasmic and nuclear protein extraction were operated according to the Thermo Scientific™ NE-PER™ Nuclear and Cytoplasmic Extraction Reagents (Thermo Fisher Scientific, Waltham, MA, USA).

### Cell Protein Extraction and Western Blotting

Cell or tissue lysates were prepared with RIPA lysis buffer (Beyotime, Shanghai, China). 15 µg proteins were loaded per lane. Proteins were transferred to a polyvinylidene fluoride membrane. After blocking with 5% nonfat milk in TBST, the membranes were incubated with the primary antibody as follows ER-α, FOXM1, Cathepsin D, β-tublin, LaminA/C (Cell Signaling Technology, Danvers, MA, USA), ER-β (AbSci, CA, USA), β-actin (HuaBio, Hangzhou, China), C-MYC and PR (Santa Cruz Biotechnology, Santa Cruz, CA, USA), then incubated with secondary antibody. Immune complexes were detected using an ECL Kit (Share Bio, Shanghai, China).

### Real-Time Polymerase Chain Reaction Analyses

Ultrapure RNA kit (ComWin Biotech, Beijing, China) was used to isolate total RNA, and 1 μg total RNA was reversed to cDNA by using the PrimerScript reverse transcription reagent kit (Vazyme Biotech, Nanjing, China). Then, the cDNA was quantified with RT-PCR by using the Power SYBR Green PCR MasterMix (Vazyme Biotech, Nanjing, China) on the ABI 7500 thermocycler (Applied Biosystems, Foster City, CA, USA) according to the manufacturer’s protocol and instrument manual. The sequences of the primers were as follows:

human *β-actin*: forward 5′-CGTGCGTGACATTAAGGAGAAG-3′,
reverse 5′-AAGGAAGGCTGGAAGAGTGC-3′;
human *ESR1*: forward 5′-GACCGAAGAGGAGGGAGAATG-3′,
reverse 5′-CAACAAGGCACTGACCATCTG-3′;
human *FOXM1*: forward 5′-ACAGCAGAAACGACCGAATC-3′,
reverse 5′-GGCAATGGCACCTTCACC-3′.

### Quantifcation of Reactive Oxygen Species

The quantification of reactive oxygen species (ROS) production was monitored by cell permeable ROS indicator 2′,7′- dichlorodihydrofuorescein diacetate (H2-DCFDA) (Sigma-Aldrich, Darmstadt, Germany). The NAC rescue assay was performed as followings: Cells were pre-incubated with 5 mM NAC for 2 h, then co-incubated with the indicated drugs and assessed for cell viability or ROS production as described above.

### Gene Silencing Using Small Interfering RNA

The cells were transfected with siRNA oligonucleotides against the following genes using the Lipofectamine 2000 (Invitrogen, Carlsbad, CA, USA), according to the manufacturer’s instructions. All siRNAs were synthesized by GenePharma (Shanghai, China). The sequences of the siRNAs were as follows:

siNC: 5′-UUCUCCGAA CGUGUCACGUTT-3′;
siFOXM1#1: 5′-GCUGGGAUCAAGAUUAUUATT-3′;
siFOXM1#2: 5′-GCCAACCGCUACUUGACAUTT-3′.

### Construction of Plasmid

Full-length complementary DNA (cDNA) of ER-α was cloned into the pCDH vector using standard protocols. All constructions were confirmed by DNA sequencing before further applications.

### Different Expressed Genes Screening

The raw data of GSE85871, GSE10061 and GSE59732 in the GEO database were analyzed by the interactive web tool, GEO2R (https://www.ncbi.nlm.nih.gov/geo/geo2r/). The GEO2R tool carried out comparisons using the limma R packages from the Bioconductor project. Statistically different expressed genes (DEGs) were defined with *P* < 0.01 and |log_2_fold change (FC)| ≥ 0.9.

### KEGG Pathway Enrichment Analysis

KEGG pathway were analyzed using the DAVID online tool (https://david-d.ncifcrf.gov/tools.jsp) at the functional level. *P* < 0.05 was set as the cut-off criterion. The top 10 enriched pathways were figured.

### Identification of AD-Associated Genes

The human genes associated with AD were acquired from the BATMAN-TCM (http://bionet.ncpsb.org.cn/batman-tcm/) database. BATMAN-TCM is an online bioinformatics analysis tool specially designed for studying the molecular mechanisms of traditional Chinese medicine (17). Score > 3 was set as the cut-off criterion.

### PPI Network Construction

The online database STRING (https://cn.string-db.org/) was used to visualize the PPIs between the statistically DEG-encoded proteins in the resultant dataset (18). We used Cytoscape software (http://www.cytoscape.org/) to visualize the PPI network obtained from the STRING database (19). In addition, the Cytoscape plugin cytoHubba was applied to analyze the hub target genes between AD and ER-positive breast cancer. The target genes were ranked by MCC algorithm.

### Transcription Factor Analysis

Transcription factors of identified modules were analyzed by the iRegulon plugin of Cytoscape (20). The iRegulon plugin was set as the default. The top five transcription factors with higher NES were listed.

### Tumor Xenograft Growth Assay

Five-week-old female BALB/c athymic nude mice purchased from Lingchang Biological Technology Co., Ltd. (Shanghai, China) were maintained and treated in accordance with established guidelines, and the protocol was approved by the Institutional Animal Care and Use Committee of Longhua hospital, Shanghai University of Traditional Chinese Medicine. All the mice were handled using aseptic procedures and allowed to acclimatize to local conditions for one week before the experimental manipulations. 1 × 10^7^ MCF7 cells with Matrigel (1:1) were injected into the right flank of each mouse. Estradiol cypionate (Selleck, Houston, Texas, USA) (1.5 mg/kg, once a week) was injected subcutaneously into each mouse one day before the injection of MCF7 cells. Mice were randomly divided into 4 groups and treated with 20% 2-hydroxypropyl-β-cyclodextrin (HPBCD), fulvestrant, AD or AD plus fulvestrant with the indicated doses. Tumor xenografts were measured with a caliper every 4 days and tumor volume was calculated as (Length × Width^2^)/2.

### Statistical Analysis

GraphPad prism8 software was used to evaluate the statistical significance of the differences among groups. Unmatched 2-tailed t-test was used to compare the parameters between groups. The level of significance was set at *P* < 0.05. For all tests, three levels of significance (*P < 0.05, **P < 0.01, ***P < 0.001) were used.

## Results

### AD Inhibits *ESR1* Transcription in ER-Positive Breast Cancer

**Figure 1:**
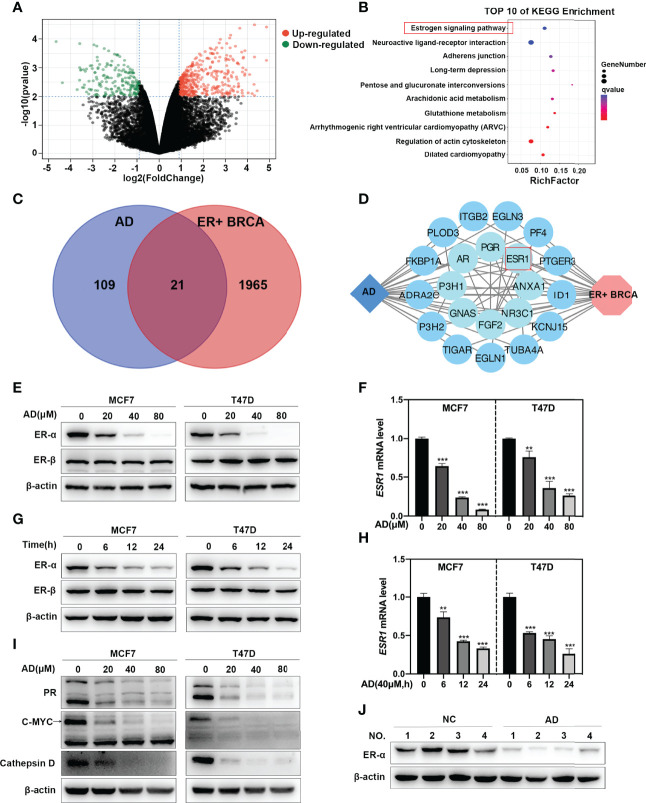
AD inhibits *ESR1* transcription in ER-positive breast cancer. **(A)** Volcano plot showing the DEGs between the control and AD treatment group from the GSE85871 dataset. **(B)** KEGG pathway enrichment analysis for the DEGs and qvalue refers to -log_10_(pvalue). **(C)** Venn plot revealing the overlapping target genes for AD against ER-positive breast cancer. Blue circle represents AD; red circle represents ER-positive breast cancer. **(D)** Network of target genes for AD against ER-positive breast cancer was built using the STRING database and Cytoscape software. Blue diamond represents AD; red hexagon represents ER-positive breast cancer; blue circles represent target genes of AD in ER-positive breast cancer and were arranged from the inner circle to the outer circle according to the degree of association. **(E)** Cells were treated with 1‰ DMSO or the indicated concentrations of AD for 24 h. Cell protein was extracted and detected by Western blotting with antibodies against ER-α, ER-β and β-actin. **(F)** The mRNA level of *ESR1* was quantified by RT-PCR (normalized to β-actin). **(G)** Cells were treated with AD (40 μM) for 0 h, 6 h, 12 h, 24 h, respectively. Cell protein was extracted and detected by Western blotting with antibodies against ER-α, ER-β and β-actin. **(H)** The mRNA level of *ESR1* was quantified by RT-PCR (normalized to β-actin). **(I)** Cells were treated with 1‰ DMSO or the indicated concentrations of AD for 24 h. Cell protein was extracted and detected by Western blotting with antibodies against PR, C-MYC, Cathepsin D and β-actin. **(J)** Proteins extracted from tumor tissues of mice treated with 20% HPBCD or AD (150 mg/kg) were detected by Western blotting with antibodies against ER-α and β-actin. (Data were presented as mean ± SD. **P < 0.01 and ***P < 0.001).

To determine the cellular responses and explore the target of AD on breast cancer cells, the GSE85871 dataset (21) was adapted to comprehensively uncover the altered pathways between the control and AD-treated group. A total of 404 genes were significantly upregulated and 194 genes were significantly downregulated (**Figure 1A**). KEGG pathway enrichment analysis of these 598 different expressed genes (DEGs) showed that the most disturbed pathway was the “estrogen signaling pathway” (**Figure 1B**).

To further ascertain whether AD affects estrogen signaling pathway in ER-positive breast cancer, we performed an integrative analysis *via* using the BATMAN-TCM database (17) and the GSE59732 dataset (22). In the BATMAN-TCM database, 130 genes were identified as AD targets (**Supplementary Table 1**). In the GSE59732 dataset, 1986 DEGs were identified in ER-positive breast cancer cell lines compared with human mammary epithelial cell lines (**Supplementary Table 2**). Venn analysis of these DEGs and AD targets from BATMAN-TCM database showed that a total of 21 genes served as potential targets of AD in ER-positive breast cancer (**Figure 1C**). Further analysis through STRING database (18) and Cytoscape software (19) showed that *ESR1*, a critical gene in the “estrogen signaling pathway”, was the key target of AD in ER-positive breast cancer (**Figure 1D**).

To verify the above findings, two ER-positive breast cancer cell lines (MCF7 and T47D) were selected to determine the ER-α level after AD treatment. As shown, AD significantly down-regulated the protein and mRNA levels of ER-α in a time- and dose-dependent manner, but did not impact the expression of ER-β in MCF7 and T47D cells (**Figures 1E–H**). Consistently, AD also suppressed the expression levels of ER-α target genes (23, 24), including PR, C-MYC and Cathepsin D (**Figure 1I**). Finally, we investigated the efficacy of AD on ER-α expression *in vivo*, as shown in **Figure 1J**, ER-α was significantly decreased after AD treatment. Taken together, these data collectively indicate that AD inhibits ER-α expression in ER-positive breast cancer both *in vitro and in vivo*.

### AD Inhibits Breast Cancer Cell Growth Through Down-Regulating ER-α Expression

**Figure 2:**
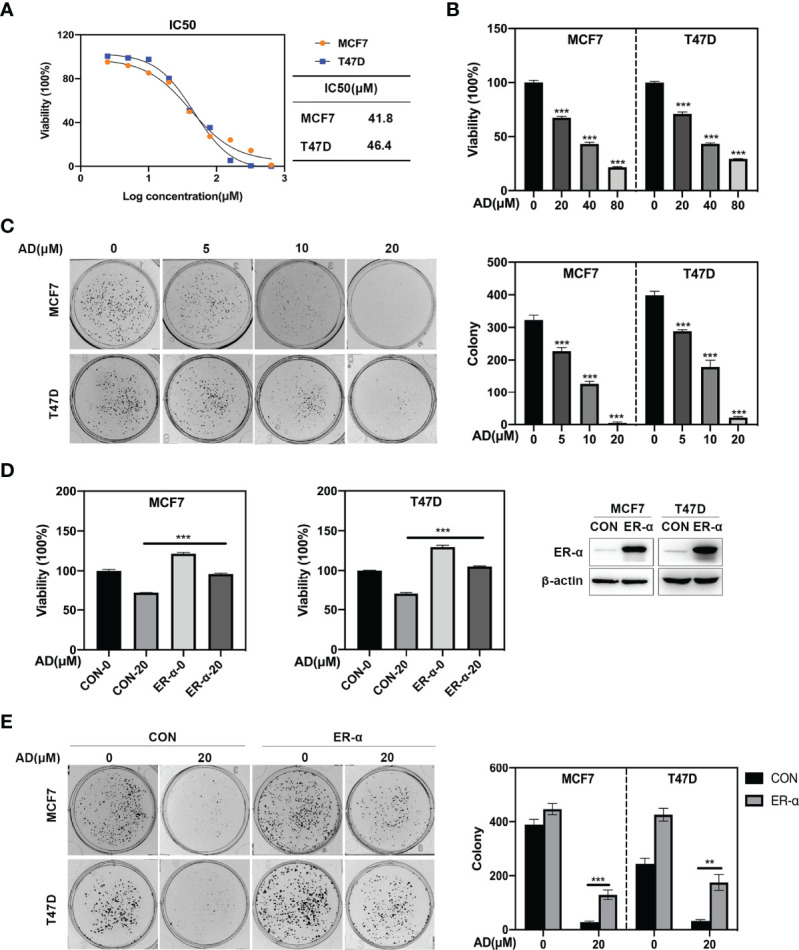
AD inhibits breast cancer cell growth through down-regulating ER-α expression. **(A)** Cells were seeded in ATPlite plates in triplicate, 3000 cells per well, cultured overnight, and treated with 1‰ DMSO or various concentrations of AD (2.5, 5, 10, 20, 40, 80, 160, 320 and 640 μM) for 72 h. The ATPlite luminescence assay was used to determine the half-maximal inhibitory concentrations (IC50) of MCF7 and T47D cells, respectively. **(B)** Cells were seeded in ATPlite plates in triplicate, 3000 cells per well, cultured overnight, and treated with 1‰ DMSO or indicated concentrations of AD for 72 h, followed by the ATPlite luminescence assay. **(C)** Representative images of three independent experiments are shown for the inhibition of colony formation by AD. **(D)** The MCF7 and T47D cells were transduced with either control vector (MCF7/CON and T47D/CON) or human ER-α-expressing lentiviruses (MCF7/ER-α and T47D/ER-α), followed by Western blotting to verify the ER-α expression. These control and ER-α-expressing cells were treated with AD (20 μM) for 72 h, followed by analyzing the cell proliferation rate using the ATPlite luminescence assay. **(E)** The MCF7/CON, MCF7/ER-α and T47D/CON, T47D/ER-α cells were treated with AD (20 μM) for 2 weeks, followed by analyzing the cell colony formation. (Data were presented as mean ± SD. **P < 0.01 and ***P < 0.001).

Given that ER-α acts as a classical oncogene to promote tumor growth (25), we firstly systematically determined the effect of AD on malignant phenotypes in ER-positive breast cancer cell lines. As shown, AD significantly inhibited the proliferation of MCF7 and T47D, and its IC50 value was 41.8 μM in MCF7 and 46.4 μM in T47D, respectively (**Figures 2A, B**). Furthermore, AD inhibited colony formation of MCF7 and T47D cells in a dose-dependent manner (**Figure 2C**). These findings indicated that AD suppressed the viability of breast cancer cell lines *in vitro*.

To further determine whether AD-induced ER-α inhibition is crucial for tumor malignant phenotypes, we transduced full-length cDNA of human ER-α into MCF7 and T47D cells and evaluated the role of ER-α on AD-treated cells. As shown in **Figures 2D, E**, ER-α overexpression significantly diminished the inhibited effect of AD on cell growth and colony formation in MCF7 and T47D cells. Together, these results indicate that AD suppresses ER-α expression to inhibit the proliferation of ER-positive breast cancer cells.

### AD Inhibits *ESR1* Transcription *via* Inducing ROS Production

**Figure 3:**
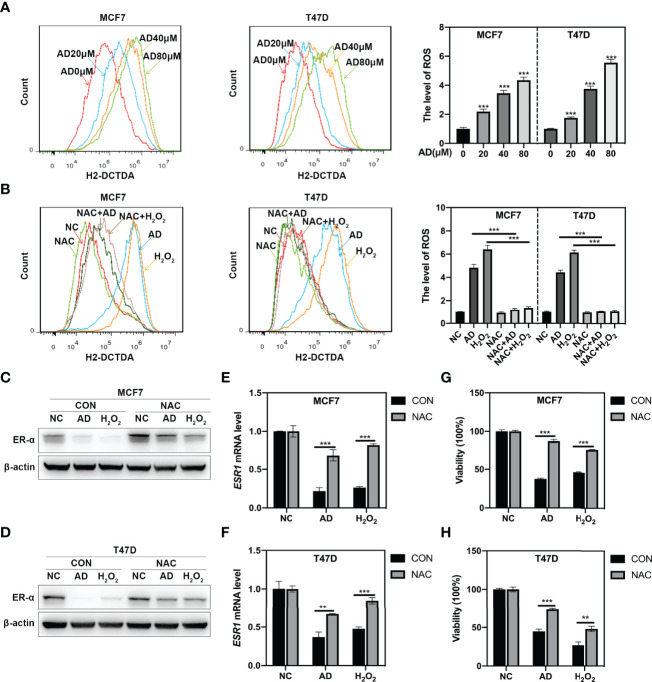
AD inhibits *ESR1* transcription *via* inducing ROS production. **(A)** AD elevated ROS levels in MCF7 and T47D cells. Cells were treated with various concentrations of AD for 24 h. The level of ROS was determined by H2-DCFDA staining and flow cytometry. **(B)** MCF7 and T47D cells were treated with AD (40 μM), H_2_O_2_ (10 μM), NAC (5 mM) alone or AD+NAC, H_2_O_2_+NAC for 24 h and subjected to H2-DCFDA staining analysis for determining the levels of ROS. H_2_O_2_ was used as a positive control. **(C–H)** N-Acetylcysteine (NAC), the classical ROS scavenger, attenuated AD-inhibited the expression of ER-α and cell growth. **(C, D)** MCF7 and T47D cells were treated with AD (40 μM) and/or NAC (5 mM) for 24 h and subjected to Western blotting for the expression of ER-α. H_2_O_2_ was used as a positive control. **(E, F)** The mRNA level of *ESR1* was quantified by RT-PCR (normalized to β-actin). **(G, H)** The cell proliferation rate was assessed using ATPlite luminescence assay. (Data were presented as mean ± SD. **P < 0.01 and ***P < 0.001).

Previous study reported that ROS could downregulate ER-α expression (26), thus we hypothesized that AD could inhibit ER-α expression through inducing ROS production. Firstly, we detected cellular ROS level through the cell permeable ROS indicator, 2′, 7′- dichlorodihydrofuorescein diacetate (H2-DCFDA) and found that AD significantly promoted ROS production in MCF7 and T47D cells (**Figure 3A**). Then, we used N-acetylcysteine (NAC), a classical ROS scavenger, to block ROS elevation and found that NAC prevented AD-induced the generation of ROS (**Figure 3B**) and markedly attenuated AD-inhibited ER-α expression both at protein (**Figures 3C, D**) and mRNA levels (**Figures 3E, F**). Furthermore, the proliferation of breast cancer cells increased after NAC treatment in AD-treated group (**Figures 3G, H**). Based on these observations, we concluded that AD induces ROS production to inhibit *ESR1* transcription and cell growth in ER-positive breast cancer cells.

### AD Suppresses *ESR1* Transcription *via* ROS-FOXM1 Axis

**Figure 4:**
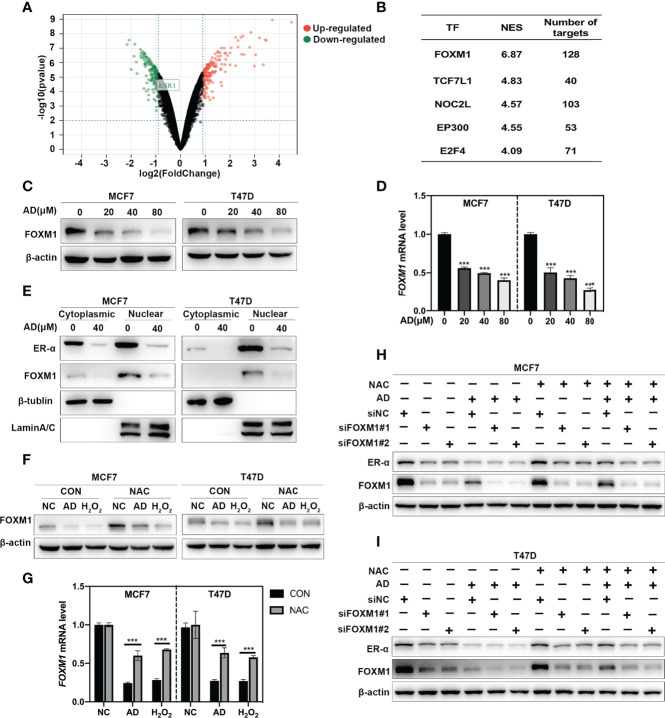
AD suppresses *ESR1* transcription *via* ROS-FOXM1 axis. **(A)** Volcano plot showing the DEGs between the control and H_2_O_2_ treatment group from the GSE10061 dataset. *ESR1* belonging to the down-regulated genes was highlighted. **(B)** The top five predicted transcription factors regulating the DEGs were listed. **(C)** Cells were treated with 1‰ DMSO or the indicated concentrations of AD for 24 h. Cell protein was extracted and detected by Western blotting with antibodies against FOXM1 and β-actin. **(D)** The mRNA level of *FOXM1* was quantified by RT-PCR (normalized to β-actin). **(E)** MCF7 and T47D cells were treated with 1‰ DMSO or AD (40 μM) for 24 h. Cytoplasmic and nuclear protein extracts were collected as described in the Materials and Methods section and subjected to Western blotting with antibodies against ER-α, FOXM1, β-tublin and LaminA/C. **(F)** NAC attenuated AD-inhibited the expression of FOXM1. MCF7 and T47D cells were treated with AD (40 μM) and/or NAC (5 mM) for 24 h and subjected to Western blotting for the expression of FOXM1. **(G)** The mRNA level of *FOXM1* was quantified by RT-PCR (normalized to β-actin). H_2_O_2_ was used as a positive control. **(H, I)** MCF7 and T47D cells were transfected with control or FOXM1 siRNA for 48 h, then treated with AD (40 μM) and/or NAC (5 mM) for 24h and subjected to Western blotting for the expression of FOXM1 and ER-α. (Data were presented as mean ± SD. ***P < 0.001).

In order to explore how AD downregulates ER-α *via* inducing ROS production, the GSE10061 dataset (27), a gene expression profiling about H_2_O_2_ treatment in MCF7 cells was used. In the GSE10061 dataset, 313 DEGs including *ESR1* were identified in the H_2_O_2_-treated group compared with that in the control group (**Figure 4A**), demonstrating that this dataset was exactly captured the molecular features of ROS-*ESR1* pathway. Given that AD decreased *ESR1* at transcription level, we clustered the altered transcription factors which might regulate *ESR1* transactivation *via* using iRegulon (20). The top five predicted transcription factors with the highest normalized enrichment score (NES) were shown in **Figure 4B**. In these transcription factors, FOXM1 gained the highest NES and could regulate 128 DEGs including *ESR1* (**Figure 4B** and **Supplementary Figure 1A**). Thus, we hypothesized that AD might suppress *ESR1* transcription *via* ROS-FOXM1 axis. To verify this hypothesis, we determined the effect of AD on FOXM1 expression. As shown, we found that AD significantly inhibited FOXM1 expression at protein and mRNA levels in a dose-dependent manner (**Figures 4C, D**). Since FOXM1 acts as a transcription factor in the nucleus to regulate *ESR1* transactivation, we detected the expression level of FOXM1 and ER-α in the nucleus upon AD treatment and found that FOXM1 and ER-α were significantly decreased in the nucleus (**Figure 4E**). Next, we used NAC to block ROS production and found that NAC markedly attenuated AD-inhibited FOXM1 expression both at protein and mRNA levels (**Figures 4F, G**). Furthermore, the rescue effect of NAC on AD-suppressed ER-α expression and cell proliferation could be alleviated by FOXM1 knockdown (**Figures 4H, I** and **Supplementary Figure 2**). These results collectively demonstrate that AD suppresses *ESR1* transcription *via* ROS-FOXM1 axis.

### AD Synergizes With Fulvestrant to Inhibit ER-α Expression and Breast Cancer Growth

**Figure 5:**
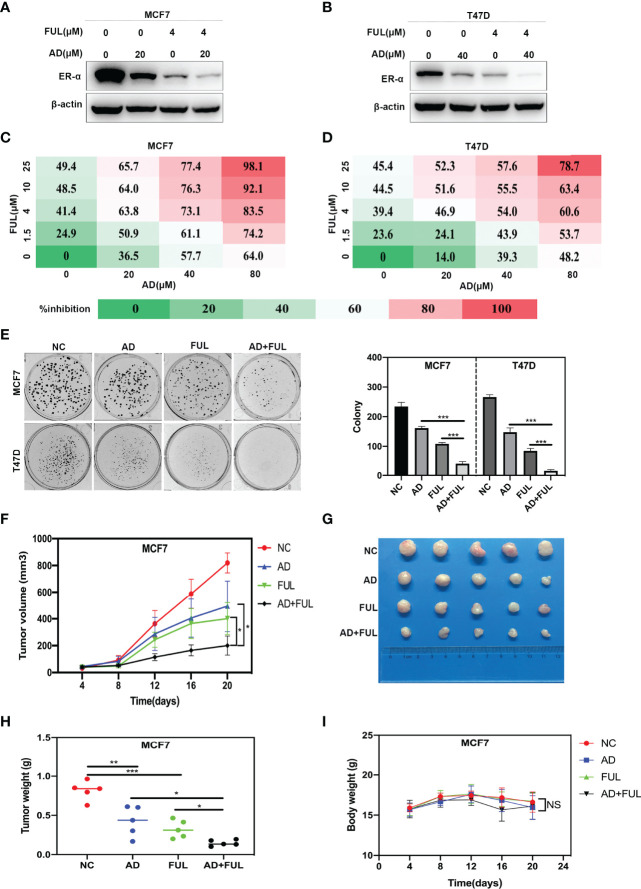
AD synergizes with fulvestrant to inhibit ER-α expression and breast cancer growth. **(A, B)** MCF7 and T47D cells were treated with AD and/or fulvestrant for 24 h and harvested for Western blotting with antibodies against ER-α and β-actin. **(C, D)** MCF7 and T47D cells were treated with escalating doses of either AD or fulvestrant for 72 h, followed by analyzing the cell proliferation rate assessed by ATPlite luminescence assay. Percent inhibition at each dose of drug is presented. **(E)** MCF7 and T47D cells were treated with AD (10 μM) and/or fulvestrant (0.1 μM) for 2 weeks, followed by analyzing the cell colony formation. **(F–I)** Nude mice bearing breast cancer xenografts with MCF7 cells were administered with 20% HPBCD, AD (150 mg/kg per day), fulvestrant (5 mg/mouse administered subcutaneously, once per week) and AD plus fulvestrant. The treatments for the nude mice were carried out on 4 days after MCF7 cells injected and lasted for 16 days. **(F)** Tumor volumes were determined by caliper measurement, and the data was converted to tumor growth curve. **(G)** Images of the tumors in each of the 4 groups at the end of experiment. **(H)** Weight of the tumors in each of the 4 groups was measured with an electronic scale immediately after the tumor was collected. **(I)** The mice weights were recorded every 4 days during the whole experiment. (Data were presented as mean ± SD. *P < 0.05, **P < 0.01, ***P < 0.001, NS denotes not significant.

Considering that AD inhibited *ESR1* transcription, we hypothesized that AD might enhance the efficacy of fulvestrant by synergistically inhibiting ER-α expression. As shown in **Figures 5A, B**, the expression of ER-α was synergistically inhibited after AD and fulvestrant combined treatment. To verify whether AD could synergize with fulvestrant to suppress cell growth, MCF7 and T47D cells were treated with AD and fulvestrant alone or in combination with escalating doses. We found that the combination of AD and fulvestrant synergistically suppressed cell proliferation (**Figures 5C, D**), with optimal concentration of AD (80 μM) and FUL (25 μM) for MCF7 and T47D cells (combination index (CI) were 0.02 and 0.4, respectively) (**Supplementary Figures 1B, C**). Moreover, AD synergized with fulvestrant to suppress the colony formation of MCF7 and T47D cells (**Figure 5E**).

Next, we determined whether AD and fulvestrant synergized to suppress ER-positive breast cancer *in vivo*. We found that, compared to AD or fulvestrant treatment alone, the combination of these two agents further inhibited tumor growth (**Figure 5F**) and reduced tumor volumes (**Figure 5G**) and tumor weight (**Figure 5H**). In addition, there were no obvious treatment-related toxicity, such as body weight loss, in single or combined groups (**Figure 5I**). Taken together, these findings demonstrate that AD synergizes with fulvestrant to inhibit ER-α expression and breast cancer growth.

## Discussion

Estrogen receptor (ER)-positive breast cancer is the main subtype of breast cancer with high incidence and mortality, which accounts for about third quarters of all breast cancer patients. In the past few years, achievements have been obtained in the development of novel anti-ER-positive breast cancer strategies and effective drugs (28). Recently, a variety of Chinese herbal extracts and isolated compounds exhibited excellent anti-tumor efficacy in breast cancer cells (29, 30). AD, one of the above compounds, has substantial anti-cancer effect in various tumors by inducing cell cycle arrest, triggering apoptosis or suppressing autophagy (13, 31, 32). In our previous study, we found that AD exhibited a broad-spectrum inhibition of proliferation in lung cancer cells *via* inducing apoptosis (15). In the present study, we found that AD inhibited ER-α expression and the proliferation of ER-positive breast cancer both *in vitro and in vivo*. In terms of mechanisms, AD suppressed *ESR1* transcription *via* inducing ROS production to down-regulate FOXM1.

ER, as one of the most successful molecular target in the history of anti-breast cancer drug discovery, determines the sensitivity and effectiveness of endocrine therapy for breast cancer (33, 34). Therefore, suppressing ER *via* endocrine therapy is recommended as the first-line treatment for ER-positive breast cancer therapy in clinic (35). Fulvestrant, an important endocrine therapy drug, inhibited ER-α expression *via* promoting the proteasomal degradation of ER-α to suppress tumor growth (36). However, the ER-α down-regulated effect of fulvestrant only happened in the nucleus and ER-α was not completely inhibited at clinically feasible dose (37, 38). Therefore, the efficacy of fulvestrant is still limited. In the present study, we found that AD suppressed ER-α expression both in the nucleus and cytoplasm and enhanced the efficacy of fulvestrant to synergistically inhibit ER-positive breast cancer growth.

FOXM1, a potent oncogene, regulates a broad spectrum of normal biological functions, including cell proliferation, cell migration, angiogenesis and cell survival (39). Kwok et al. reported that thiostrepton, a FOXM1 inhibitor, exerted great antitumor effect by inducing cell cycle arrest and caspase-dependent apoptosis in MCF7 cells (40). In addition, FOXM1 and ER-α were closely related in breast cancer (41). FOXM1 was reported to regulate ER-α expression through binding to the two forkhead response elements located at the proximal region of the *ESR1* promoter (23). However, the up-stream of FOXM1-ER-α axis is still unknown. Previous studies reported that ROS could significantly regulate oxidative stress related genes including *FOXM1* (42, 43). In the present study, through bioinformatic analysis and experiments, we found that ROS served as an up-stream signal of FOXM1-ER-α axis and down-regulated the expression of FOXM1 at both protein and mRNA levels. Furthermore, pre-treatment with the antioxidant NAC prevented AD-induced generation of ROS and significantly attenuated AD-inhibited FOXM1 expression. Based on these findings, we demonstrated a novel mechanism that AD induced ROS production to down-regulate FOXM1-ER-α axis, thereby inhibiting ER-positive breast cancer growth.

**Figure 6:**
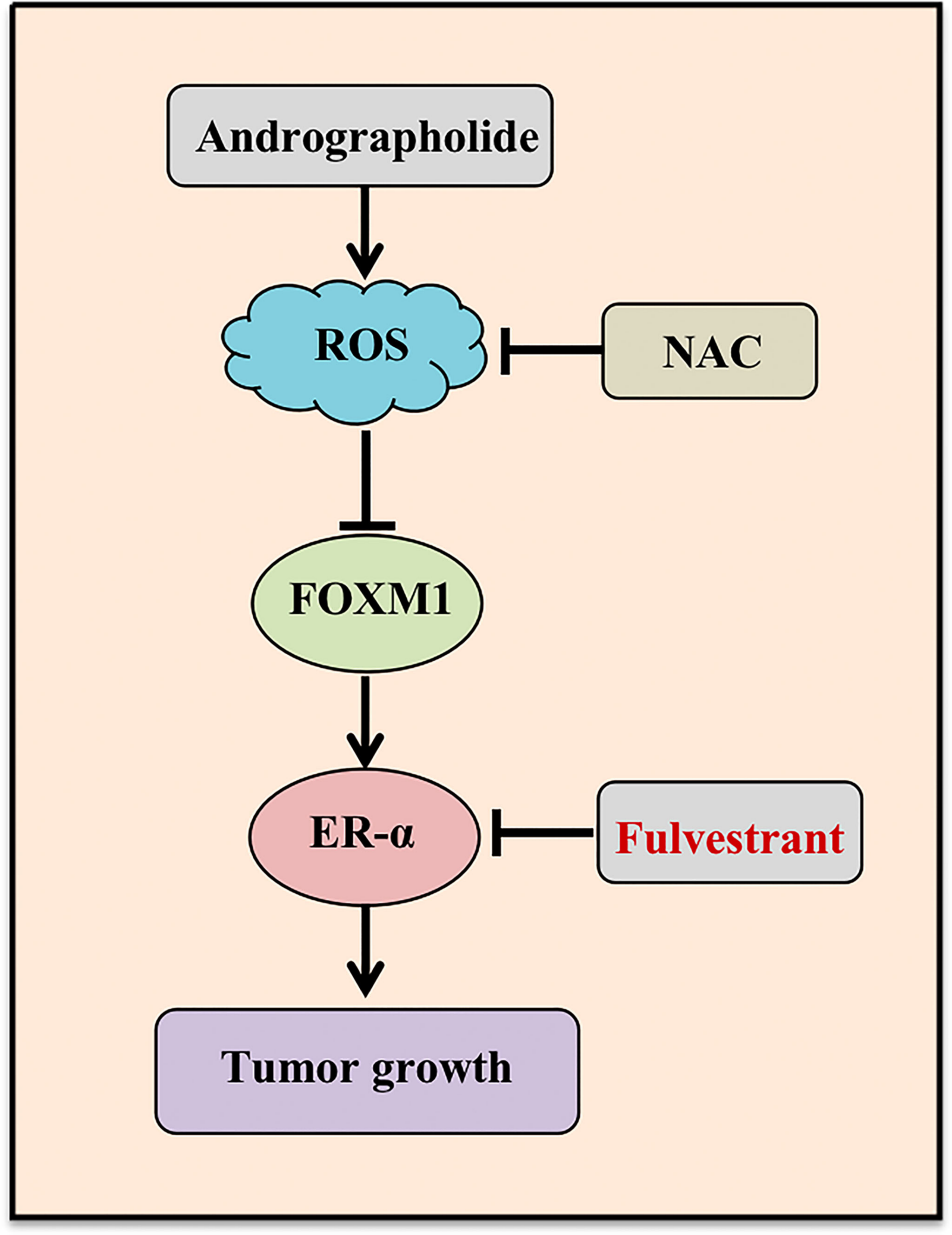
Working model. AD suppressed *ESR1* transcription through ROS-FOXM1 axis and synergized with fulvestrant to inhibit ER-α expression and breast cancer growth.

In conclusion, our study highlighted a pivotal role of AD in suppressing the tumor progression of ER-positive breast cancer both *in vitro and in vivo*, and discovered a novel mechanism of AD down-regulating the expression of ER-α in ER-positive breast cancer and enhancing fulvestrant efficacy (**Figure 6**). These findings suggested that AD is a potential therapeutic agent to ameliorate outcomes for breast cancer patients.

## Data Availability Statement

The datasets presented in this study can be found in online repositories. The names of the repository/repositories and accession number(s) can be found in the article/**Supplementary Material**.

## Ethics Statement

The animal study was reviewed and approved by Animal Experimental Ethics Committee of Longhua hospital, Shanghai University of Traditional Chinese Medicine.

## Author Contributions

TX, YJ, and LJ conceived the general framework of this study and designed the experiments. TX, YJ, and SY performed the experiments and drafted the manuscript. LC and LZ provided technical or material support. XC, WZ and BX performed statistical analyses. LJ supervised this study. All authors contributed to the article and approved the submitted version.

## Funding

This work was supported by the National Natural Science Foundation of China (Grant Nos. 81820108022 and 82003297), Shanghai Frontiers Science Center of Disease and Syndrome Biology of Inflammatory Cancer Transformation (2021KJ03-12), Innovation Program of Shanghai Municipal Education Commission (2019-01-07-00-10-E00056), ChenGuang project supported by Shanghai Municipal Education Commission and Shanghai Education Development Foundation (19CG49).

## Conflict of Interest

The authors declare that the research was conducted in the absence of any commercial or financial relationships that could be construed as a potential conflict of interest.

## Publisher’s Note

All claims expressed in this article are solely those of the authors and do not necessarily represent those of their affiliated organizations, or those of the publisher, the editors and the reviewers. Any product that may be evaluated in this article, or claim that may be made by its manufacturer, is not guaranteed or endorsed by the publisher.
