# Supplementary material for: Andrographolide Inhibits ER-Positive Breast Cancer Growth and Enhances Fulvestrant Efficacy via ROS-FOXM1-ER-α Axis
Source: Front Oncol. 2022 May 9;12:899402. doi: 10.3389/fonc.2022.899402 (PMC9124841; doi:10.3389/fonc.2022.899402)
Supplement: Supplementary file 1 [file DataSheet_1.pdf]

Supplementary Material

1 Supplementary Figures and Tables

1.1 Supplementary Tables

Supplementary Table 1 List of AD-target genes via using the BATMAN-TCM database.

Supplementary Table 2 List of breast cancer-target genes via using the GSE59732 dataset.

1.2 Supplementary Figures

Xu et al. Supplementary Fig. 1

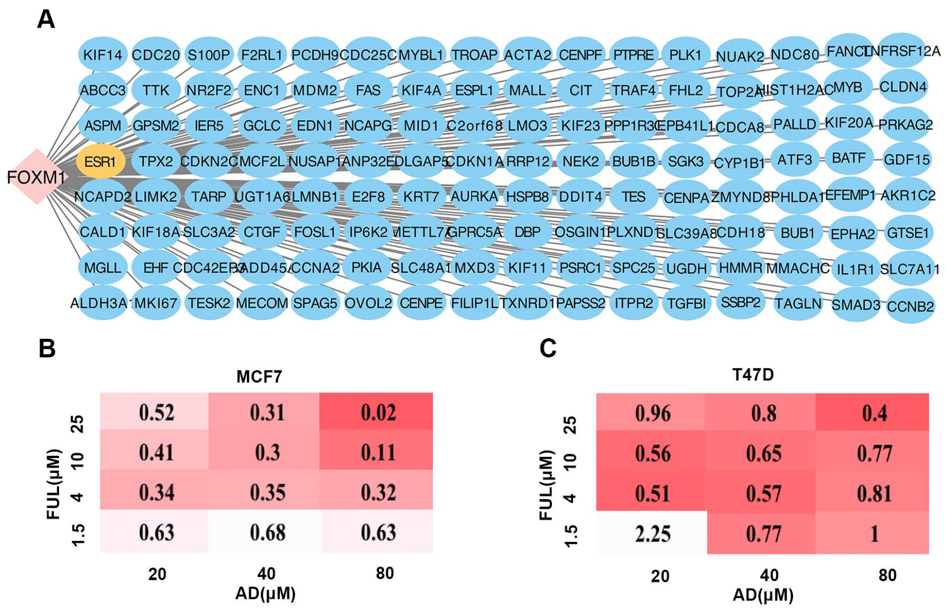

Supplementary Figure 1. Network of target genes regulated by FOXM1 and the combination index (CI) for MCF7 and T47D cells. (**A**) Network of target genes regulated by FOXM1. (**B**) The combination index (CI) for MCF7. The optimal combination index (CI) for MCF7 cells was 0.02. (**C**) The combination index (CI) for T47D. The optimal combination index (CI) for T47D cells was 0.4.

Xu et al. Supplementary Fig. 2

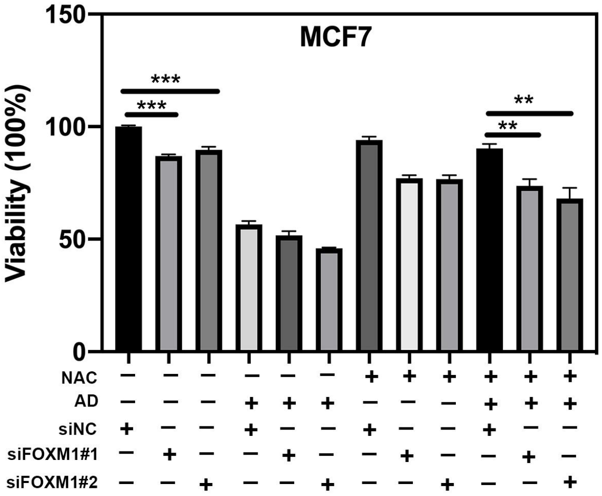

Supplementary Figure 2. The effect of AD and/or NAC on cell proliferation in FOXM1 knockdown cells. MCF7 cells were transfected with control or FOXM1 siRNA for 48 h, then treated with AD (40 μM) and/or NAC (5 mM) for 48h, followed by analyzing the cell proliferation rate assessed by ATPlite luminescence assay. (Data were presented as mean ± SD. $**P < 0.01$, $***P < 0.001$).
